# Emerging Enhancement and Regulation Strategies for Ferromagnetic 2D Transition Metal Dichalcogenides

**DOI:** 10.1002/advs.202300952

**Published:** 2023-05-13

**Authors:** Fan Yang, Ping Hu, Fairy Fan Yang, Bo Chen, Fei Yin, Ruiyan Sun, Ke Hao, Fei Zhu, Kuaishe Wang, Zongyou Yin

**Affiliations:** ^1^ School of Metallurgy Engineering State Local Joint Engineering Research Center for Functional Materials Processing Xi'an University of Architecture and Technology Xi'an 710055 China; ^2^ Research School of Chemistry The Australian National University Canberra ACT 2601 Australia

**Keywords:** 2D materials, diluted magnetic semiconductors, enhancement strategies, ferromagnetic, transition metal dichalcogenides

## Abstract

Two‐dimensional transition metal dichalcogenides (2D TMDs) present promising applications in various fields such as electronics, optoelectronics, memory devices, batteries, superconductors, and hydrogen evolution reactions due to their regulable energy band structures and unique properties. For emerging spintronics applications, materials with excellent room‐temperature ferromagnetism are required. Although most transition metal compounds do not possess room‐temperature ferromagnetism on their own, they are widely modified by researchers using the emerging strategies to engineer or modulate their intrinsic properties. This paper reviews recent enhancement approaches to induce magnetism in 2D TMDs, mainly using doping, vacancy defects, composite of heterostructures, phase modulation, and adsorption, and also by electron irradiation induction, O plasma treatment, etc. On this basis, the produced effects of these methods for the introduction of magnetism into 2D TMDs are compressively summarized and constructively discussed. For perspective, research on magnetic doping techniques for 2D TMDs materials should be directed toward more reliable and efficient directions, such as exploring advanced design strategies to combine dilute magnetic semiconductors, antiferromagnetic semiconductors, and superconductors to develop new types of heterojunctions; and advancing experimentation strategies to fabricate the designed materials and enable their functionalities with simultaneously pursuing the upscalable growth methods for high‐quality monolayers to multilayers.

## Graphical abstract

The existence of conventional magnetic ions may not be necessary for the magnetism of 2D transition metal dichalcogenides (TMDs), but the local magnetic moment can effectively regulate the magnetism. Recent enhancement approaches for introduction of magnetism into 2D TMDs mainly use element doping, vacancy defects, heterostructures, phase modulation, adsorption, and also electron irradiation induction, O plasma treatment, etc.

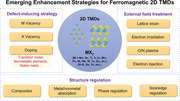

## 1 Introduction

Since the successful exfoliation of graphene by Geim's group at the University of Manchester in 2004, researchers have initiated extensive research on a wide range of 2D materials represented by graphene.^[^ ^1^ ^]^ Due to quantum confinement effects, atomically ultrathin 2D materials with a few nanometers thick have electrons that can only move freely in the 2D plane. As a typical representative of 2D materials, 2D transition metal disulfides (TMDs) have completely different energy band structures and electrical properties involving atomic‐level thickness, direct bandgaps, good electrical and mechanical properties, etc., so they have a extensive application prospect in spintronics.^[^ ^1^, ^2^ ^]^

Unfortunately, most 2D TMDs are nonmagnetic, which limits their application in the field of spintronics. However, it has been found in simulations that 2D TMDs can be made magnetic by modulation such as doping, hydrogenation, and building zigzag edges, and many experimental results have proved this idea.^[^ ^3^ ^]^ Under the continuous experiments of researchers, some researchers found that the magnetic behavior on 2D TMDs can be manipulated by applying elastic strain.^[^ ^4^ ^]^ The strain can weaken the metal–ligand bonding of 2D TMDs, altering the exchange interactions between the local spin moments and leading to significant mechanical–magnetic coupling. More excitingly, some researchers have found that the pristine VX_2_ (X = S, Se) is also magnetic.^[^ ^5^ ^]^ This gives hope for the study of the magnetic properties of 2D transition metal disulfides.

**Scheme 1:**
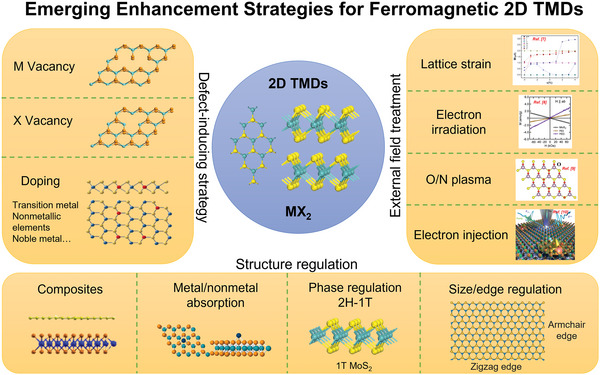
Emerging enhancement strategies for ferromagnetic 2D transition metal dichalcogenides. Reproduced with permission.^[^ ^7^ ^]^ Copyright 2017, Springer US. Reproduced with permission.^[^ ^8^ ^]^ Copyright 2016, American Institute of Physics. Reproduced with permission.^[^ ^9^ ^]^ Copyright 2022, Springer US. Reproduced with permission.^[^ ^10^ ^]^ Copyright 2022, American Chemical Society.

For better applications in the field of nanoelectronics, more precise and flexible control of the magnetic properties of 2D TMDs is required. In terms of spintronic devices, dilute magnetic semiconductors have a very wide range of potential applications. Studying the magnetism of dilute magnetic semiconductors can lay the foundation for their further applications in spintronic devices. Therefore, how to introduce magnetism in such material systems as transition metal disulfides and induce them to produce room‐temperature ferromagnetism with high magnetization strength is the key to their applicability in spintronics devices. This research area has become a hot spot for the development of spintronics, which is of great importance to the development of spintronics.^[^ ^6^ ^]^ Herein, we summarize the emerging enhancement strategies for ferromagnetic 2D TMDs (**Scheme** **1**). First, we introduce the recent enhancement approaches to induce magnetism in 2D TMDs, mainly using transition metal doping, vacancy defects, composite of heterostructures, phase modulation, etc. Subsequently, the research results of these methods for the introduction of magnetism into 2D TMDs are compressively summarized and constructively discussed. Lastly, we analyze the research gaps and put forward our perspective on the challenges and future research directions in the fields of ferromagnetic regulation for 2D TMDs.

## 2 2D Transition Metal Dichalcogenides

Since the discovery that 2D materials of atomic‐scale thickness can be obtained by mechanical exfoliation, research on 2D materials has flourished, and their excellent properties in areas such as optoelectronics in particular have intensified the interest in them. The common chemical formula for 2D TMDs is MX_2_, where M is a transition metal element (M = Mo, Sc, Ti, V, Cr, Mn, Fe, Ni, Nb, W, Ta, Sn, etc.) and X is a sulfur element (X = S, Se, Te, etc.). In 2D TMDs, the bulk is usually a layered structure with atoms within the layers connected by strong covalent bonds and relatively weak van der Waals bonds between the layers.^[^ ^11^ ^]^ When the bulk material is thinned to the thickness of a single atomic layer, a single layer or a few layers of 2D material can be obtained. Due to the quantum confinement effect, 2D materials with only atomic‐level thickness possess excellent properties different from 3D materials. 2D TMDs are a very promising class of low‐dimensional materials with variable energy band structure and a variety of emerging properties, including tunable bandgaps, high electron mobility, high exciton binding energy, excellent thermal stability, and flexibility.^[^ ^2^, ^3^, ^4^, ^6^ ^]^

For decades, the Mermin–Wagner theorem has been an “empirical criterion” for understanding 2D magnetism.^[^ ^12^ ^]^ The theorem states that at any nonzero temperature, a long‐range magnetic sequence, either ferromagnetic or antiferromagnetic, cannot exist in a truly isotropic 2D system because its magneton excitation gap cannot resist the thermal perturbations that break the spin sequence. In recent years, the emergence of magnetic van der Waals crystals has broken the original knowledge: it has been shown that even a small uniaxial magnetic anisotropy can open a large magneton excitation gap, thus breaking the constraint of the Mermin–Wagner theorem and indicating that 2D magnetism exists at a finite Curie temperature.

Strong magnetic anisotropy in 2D materials can suppress thermal perturbation effects and produce long‐range magnetic coupling, resulting in stable magnetic ordering.^[^ ^12^, ^13^ ^]^ It has been reported in the literature that magnetic ordering has been detected in 2D materials with long spin relaxation times and spin diffusion lengths.^[^ ^5^, ^14^ ^]^ 2D TMDs with strong spin–orbit coupling can generate large magnetic anisotropy and band splitting to suppress thermal perturbation effects and thus maintain long‐range magnetic coupling. These materials have intrinsic long‐range magnetic order, and their magnetic properties have a clear dependence on the number of layers, and when the thickness of the bulk system is reduced to the monolayer limit, they usually exhibit a significantly different magnetic behavior from the bulk and few‐layer samples.^[^ ^15^ ^]^ The magnetic properties of the bulk and few‐layer samples are very dependent on the number of layers.

## 3 Ferromagnetic Source of 2D TMDs

**Figure 1:**
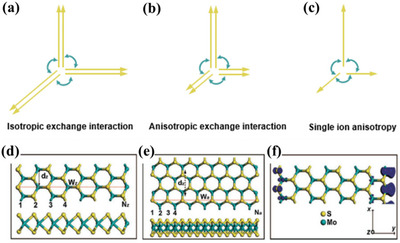
Schematic diagram of a) isotropic exchange interaction, b) anisotropic exchange interaction, and c) single ion anisotropy. d) Zig‐zag edge and e) armchair edge of monolayer MoS_2_ material, and f) spin density distribution at the zig‐zag edge. Reproduced with permission.^[^ ^17^ ^]^ Copyright 2008, American Chemical Society.

Magnetism is one of the most fundamental properties of materials and is determined by the exchange interactions between spins which align the magnetic moments along the preferred direction. In the early stages, many efforts focused on the defect or boundary‐induced magnetism, which is called externally induced magnetism by this method, magnetism can be introduced into nonmagnetic materials. From a theoretical point of view, two basic concepts are proposed to explain this fascinating magnetic phenomenon, namely, exchange interactions and spin–orbit coupling.^[^ ^12^, ^13^, ^16^ ^]^ The interactions between exchange interactions, spin–orbit coupling, and the Seeman effect are the essence of magnetic research. Together, they explain the origin of spin alignment, orbital moments, and magnetocrystal anisotropy, and the effect of external fields on these quantities where interatomic/interelectronic exchange interactions are central to the phenomenon of long‐range magnetic ordering. The schematic diagram of isotropic exchange interaction, anisotropic exchange interaction, and single ion anisotropy is shown in **Figure** **1**a–c.

Magnetic properties in 2D materials are related to anisotropy and superexchange interactions. Magnetic anisotropy in crystals arises from several microscopic mechanisms. 1) Magnetic dipole interactions, which do not contribute to cubic crystalline systems and cause anisotropy in noncubic crystals but are generally not the main contribution. 2) Spin–orbit interactions, where the spin–orbit coupling is the main source of magnetic anisotropy in d‐electron transition metals. 3) Crystal field and spin–orbit coupling together, in 4f‐electron rare earth elements, contribute significantly to magnetic anisotropy. The magnetic crystal anisotropy is generated by the interaction between the spontaneous magnetization strength and the lattice, and the change of temperature leads to the change of spontaneous magnetization strength will therefore affect the magnetic crystal anisotropy, which is more dependent on temperature compared to the spontaneous magnetization strength. Magnetic anisotropy tends to be stronger in low‐dimensional materials or at surfaces and interfaces than in bulk materials and can be modulated by external electric fields, strain, etc.^[^ ^15^, ^18^ ^]^ Magnetic crystal anisotropy, plays an important role in magnetic recording and magnetic storage, and increasing the magnetic anisotropy performance can improve the stability and storage density of storage cells. Due to their strong anisotropy, stable spontaneous magnetization can exist in these monolayer 2D materials. The anisotropy comes mainly from the strong spin–orbit coupling of heavy atoms. Asymmetric lattice distortions or an increase in the density of states near the Fermi energy level may also lead to anisotropy. The relationship between anisotropy and the strength of stable spontaneous magnetization is discussed based on spin‐wave theory and the Mermin–Wagner theorem.^[^ ^12^, ^19^ ^]^

The origin of magnetism/magnetic tuning in transition metal disulfides is mainly via doping of transition metals, vacancy defects, and adsorption. The most fundamental reason for the change process of magnetic/magnetic properties is the alteration of the relevant factors affecting the electron spin. This is indirectly reflected in the different doping elements, their concentration, doping sites (configuration), the way the doping atoms act (single atoms, paired atoms), the distance between the doping atoms, the position and number of vacancy defects, and defects in the adsorption medium. Therefore, its magnetic properties can be effectively controlled by external perturbations, such as electric field, strain, doping, chemical functionalization, and stacking engineering. According to related research, depending on the growth process, edge‐terminated or basal plane‐terminated transition group metal disulfides can be synthesized.^[^ ^15^, ^20^ ^]^ Edge‐terminated atoms play a very important role in hydrodesulfurization catalysis, and in addition, these edge atoms may exhibit unique magnetic and electrical properties that are different from those of crystals. Due to changes in coordination, the edge atoms on the surface of the cross‐section do not always remain in bulk stoichiometric equilibrium Therefore, various types of reconfigurations as well as nonuniform spin distributions may occur. There are also calculations showing that edges with different termination orientations remain in different magnetic ground states, with armchair edges being stable in the nonmagnetic (or substable magnetic) state and sawtooth edges being stable in the magnetic ground state with a net magnetic moment.^[^ ^21^ ^]^ From this perspective, in the presence of serrated edges, magnetic properties can be observed in transition group metal disulfide nanoribbons, nanocrystalline thin films, and even in the bulk limit, as long as the average grain size is small enough. At the same time, they become nonmagnetic when the transition group metal disulfide thin films exceed a certain thickness. In conclusion, jagged edges, defects, or doping induction can enable transition group metal disulfides to acquire weak magnetic properties and have a wide range of promising applications for integrated magnetic devices.

## 4 Defect‐Inducing Strategy

Due to their unique mechanical, electronic, magnetic, and optical properties, transition metal sulfides are widely used in optoelectronic devices, batteries, and novel spintronics. Among them, the application of novel spintronics requires materials with excellent room‐temperature ferromagnetism. Although transition metal sulfides have the advantages of rich and varied physical properties, small size, and low power consumption, most of the pristine transition metal compounds do not possess room‐temperature ferromagnetism, which limits their application in spintronic devices. Therefore, in practical applications, to induce magnetic properties, defect engineering methods are often used to modify transition metal compounds to design or tune their inherent properties. Common modification methods include vacancy and doping, which will be described in detail below.

### 4.1 Vacancy Strategy

According to the theory of ferromagnetism, the condition for the creation of magnetic moments is the presence of unpaired electrons in the system. For example, researchers have proposed two different edge structures, zig‐zag edge and armchair edge, when studying the electrical and magnetic properties of transition metal sulfides (Figure 1d–f).^[^ ^17^ ^]^ The spin‐polarized and nonspin‐polarized calculations for both edges show that only the zig‐zag edge can bring ferromagnetism to the material. This is due to the unsaturated covalent bonds of atoms at the edge of the zig‐zag structure, and there are unpaired electrons around them, which can provide the magnetic moment for the system, making the material exhibit certain ferromagnetism.^[^ ^17^ ^]^ In addition, studies have also shown that vacancies in the material can provide unsaturated bonds, which can significantly change the charge distribution and induce ferromagnetism. Based on this, researchers often introduce Mo, S, Se, and other vacancies to achieve magnetic induction or regulation of TMDs.

#### 4.1.1 M Vacancy

**Figure 2:**
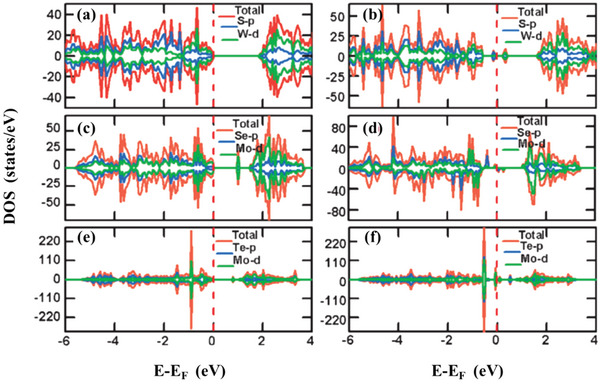
Total DOS and corresponding PDOS of sample: a) monolayer WS_2_ with one S vacancy, b) monolayer WS_2_ with one W vacancy, c) monolayer MoSe_2_ with one Se vacancy, d) monolayer MoSe_2_ with one Mo vacancy, e) monolayer MoSe_2_ with one Te vacancy, and f) monolayer MoTe_2_ with one Mo vacancy. (The vertical dashed line represents the Fermi level.) Reproduced with permission.^[^ ^22^ ^]^ Copyright 2011, Royal Society of Chemistry.

Because the chemical formula of TMDs is MX_2_, two kinds of vacancies can theoretically be generated, one is the absence of M atoms, and the other is the absence of X atoms, namely M vacancies and X vacancies. Ma et al.^[^ ^22^ ^]^ conducted a first‐principles analysis of the magnetism of transition metal sulfides in 2011. The results show that only M vacancies can change the magnetic moment and thus affect the magnetic properties of TMDs. This is due to the presence of anion vacancy, the density of states (DOS) remains spin unpolarized and the total magnetic moment of the system is still zero. The introduction of X vacancies will not destroy the original semiconductor structure of the material, but can only control the bandgap. Calculations show that Mo vacancies introduce strong spin polarization and lead to the formation of local moments in the monolayer MoSe_2_, thereby introducing magnetism, and its DOS and PDOS are shown in **Figure** **2**. Kanoun^[^ ^23^ ^]^ used the first principles to study the effect of different vacancy defects on the magnetic properties of MoTe_2_. The study showed that only the introduction of Mo vacancies made the material show the magnetic order of the magnetic moment. The magnetic moment is generated by the adjacent Te and Mo atoms around the Mo vacancy. Numerous studies have proved that the introduction of vacancy defects in TMDs is best performed on M to obtain magnetic characteristics. If vacancies are to be introduced into X, other modification measures may be required to induce better magnetic properties.

#### 4.1.2 X Vacancy

**Figure 3:**
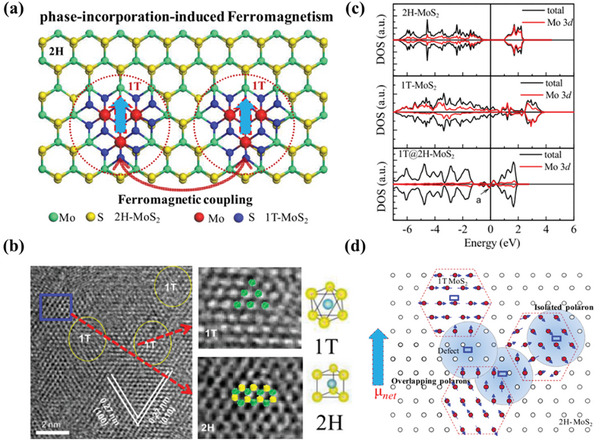
a) Schematic diagram of the phase‐incorporation strategy, b) HRTEM image of 1T@2H‐MoS_2_ nanosheets, c) DOS images of 2H‐MoS_2_, 1T‐MoS_2_, and 1T@2H‐MoS_2_ nanosheets, and d) schematic diagram of the magnetopolaron. Reproduced with permission.^[^ ^24^ ^]^ Copyright 2015, American Chemical Society.

Although more studies have shown that M vacancies have a more significant effect on the magnetic properties of TMDs, X vacancies can also work together with other modification methods to tune the magnetic properties. For example, the introduction of S or Se vacancies in the experiment can effectively form unsaturated covalent bonds, and at the same time interact with the original M^4+^ ions to induce and regulate ferromagnetism. Cai et al.^[^ ^24^ ^]^ adopted a phase‐incorporation strategy (**Figure** **3**a), introducing 1T‐MoS_2_ into 2H‐MoS_2_ nanosheets to prepare 1T@2H‐MoS_2_, which effectively combines the semiconductor properties of 2H‐MoS_2_ with the magnetic properties of 1T‐MoS_2_ (Figure 3b). The study shows that a dramatic ferromagnetic response at room temperature can be observed with the magnetic moment increasing from 0.02 µ_B_/Mo to 0.25 µ_B_/Mo. Calculations show that the enhancement of ferromagnetism originates from the exchange interaction between S vacancies and Mo^4+^ ions. The spins of the S vacancies align with the spins of nearby Mo ions in the 1T phase, providing a magnetic moment that generates an effective magnetic field and activates ferromagnetic interactions between Mo ions within the polaron radius. Its DOS diagram is shown in Figure 3c, and the mechanism of action is shown in Figure 3d. Studies have also shown that dopants can also interact with vacancies to jointly promote magnetic generation.

Han et al.^[^ ^25^ ^]^ systematically investigated the effects of Mn doping and S vacancies on the magnetism of monolayer MoS_2_ using a hybrid functional approach. The results show that the ferromagnetism of the designed target material is significantly enhanced relative to that of monolayer MoS_2_. The analysis shows that the ferromagnetism of monolayer MoS_2_ with Mn‐V_S_ clusters originates from the ferromagnetic orientation between the spins at Mn and the induced spins at the host atoms, it also originates from the contribution of the localized electron polarization of S vacancies. Notably, the effect of these vacancy defects on magnetism often depends on their symmetry, repetition periodicity, and position relative to the nanoribbon edge. With the development of computational science, researchers have been able to design and study vacancies in specific locations. Lin et al.^[^ ^26^ ^]^ used density functional theory to study the effect of different vacancies on monolayer MoSe_2_. The results show that MoSe_2_ introduced into V_Se_ is nonmagnetic despite the low formation energy of V_Se_. This is because the Mo atoms around the vacancies form symmetric Mo—Mo bonds and do not generate unsaturated dangling bonds. While the corresponding V_Mo_ does not have a dominant Fermi level, the Se atoms around V_Mo_ contain dangling bonds and unpaired electrons, which lead to electron rearrangement and generate a large magnetic moment of 3.939 µ_B_.

### 4.2 Doping Strategy

Elemental doping is a common modification method for 2D TMDs, which can control their electrical, optical, and magnetic properties. Regulating the magnetic properties of 2D transition metal sulfides by doping makes them more promising for applications in spintronics and valley electronics. By years of investigating, doping elements for 2D TMDs have been extended extremely wide, including transition metals, noble metals, rare earth elements, nonmetallic elements, and even including the p/n‐type doping elements. In this section, we discuss the mechanism and magnetic properties of the above‐mentioned types of doping.

#### 4.2.1 Transition Metal Element Doping

Transition metal element doping has been proven to be an effective means to introduce magnetism into 2D TMDs and has been widely used in many traditional magnetic semiconductors. Exploring the impact of transition metal doping on the magnetic properties of TMDs has the potential to advance the use of 2D TMDs in spintronics, and to introduce a new category of 2D magnetic materials for future electronic devices. Based on this, this section provides an in‐depth discussion on the doping of main transition metal elements such as “Fe, Mn, Co, Ni, V, Cr, Cu”.

**Figure 4:**
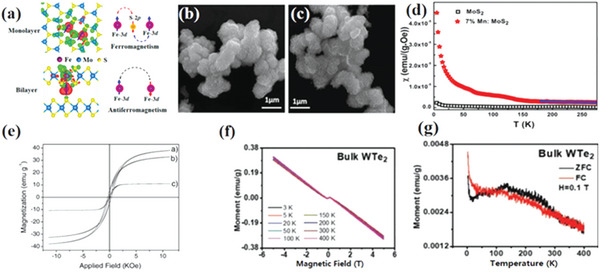
a) Magnetic properties of Fe‐doped monolayer and multilayer MoS_2_. Reproduced with permission.^[^ ^28^ ^]^ Copyright 2015, American Chemical Society. b) Typical SEM images of undoped and c) Mn‐doped MoS_2_ nanostructures. d) The temperature dependence of susceptibility of undoped MoS_2_ and 7% doped MoS_2_ under 500 Oe electric field (*χ*–*T* curve). Reproduced with permission.^[^ ^30^ ^]^ Copyright 2016, American Institute of Physics. e) M–H curve of nanocrystals after reduction for 24 h. Ni/WS_2_.^[^ ^31^ ^]^ Reproduced with permission.^[^ ^31^ ^]^ Copyright 2007, Elsevier. f) M–T curve of pure WTe_2_. g) Zero field cooling (ZFC) and field cooling (FC) M–T curves of pure WTe_2_ under 0.1 T magnetic field. Reproduced with permission.^[^ ^32^ ^]^ Copyright 2021, Wiley‐VCH.

*Fe Doping*: Fe doping has been extensively investigated and it has been established that it generally controls the magnetic characteristics of 2D TMDs. Kang et al.^[^ ^27^ ^]^ studied the influence of Fe‐doped monolayers MoS_2_ and WS_2_ on their magnetic properties. Studies have shown that both Fe‐MoS_2_ and Fe‐WS_2_ obtained by doping can induce similar ferromagnetic behavior, and n‐type materials can be obtained. However, the two exhibit different magnetic properties at room temperature, Fe‐MoS_2_ is ferromagnetic, while Fe‐WS_2_ is paramagnetic. Further studies show that isolated and paired Fe atoms exhibit different effects on magnetic properties. Isolated Fe atoms can induce ferromagnetic behavior of Fe‐MoS_2_ and Fe‐WS_2_, but the magnetic moment of paired Fe atoms only disappears in Fe‐WS_2_. As S is more electronegative than Fe, S is expected to pull negative charge from the valence states of neighboring Fe, which unpairs a d‐orbital electron bound to a Fe atom, granting it a net magnetic moment. This behavior can also be described by this principle. The amount of this charge transfer in the Fe‐doped system is examined using the Bader charge. Similar nonzero magnetic moments can be observed in isolated Fe atoms. In contrast to Fe:MoS_2_, when Fe is doped, the charge transfer to Fe atoms in Fe:WS_2_ is much less. This explains why the Fe atom in Fe lacks a magnetic moment in WS_2_. This indicates that the magnetic properties of Fe‐WS_2_ and Fe‐MoS_2_ are different. Further calculations of first‐principles show that it may be that paired Fe atoms generate a more accumulated charge by pulling away from the Fe atoms in Fe‐WS_2_, resulting in a weaker magnetic moment of paired Fe atoms in Fe‐WS_2_ than in Fe‐MoS_2_. In addition, Shu et al.^[^ ^28^ ^]^ found that iron dopants prefer ferromagnetic coupling in single‐layer MoS_2_ and antiferromagnetic coupling in double‐layer and multilayer MoS_2_ (**Figure** **4**a), which shows the dependence of magnetic exchange coupling on layers. Therefore, Xia et al.^[^ ^29^ ^]^ prepared Fe‐doped MoS_2_ nanosheets. It has been shown that the S‐vacancy alters the distribution of electron clouds around it, causing strong interactions between spin impurities. As a result, the doped Fe atoms are bound by the S‐vacancies, forming a number of bound magnetopolarons and giving Fe‐MoS_2_ long‐range ferromagnetism through ferromagnetic coupling.

*Mn Doping*: As a commonly used doped transition metal element, it is necessary to study the influence of Mn on the magnetic properties in depth. At present, a lot of work has been carried out on Mn doping. Studies have also shown that vacancy defects and Mn doping can produce a good and synergistic effect, together to produce magnetic regulation. Wang et al.^[^ ^30^ ^]^ found that the doping of Mn introduced both paramagnetic and ferromagnetic components. Figure 4b,c shows SEM images of undoped MoS_2_ and 7% Mn‐doped MoS_2_ nanostructures. It can be observed from the figure that the nanostructure is a flower shape composed of thin nanosheets, and the morphology is not obvious after doping. As seen from Figure 4d, the magnetic susceptibility of 7% Mn doped MoS_2_ is significantly higher than that of undoped MoS_2_. Some studies have shown that Mn can also be doped with other atoms to improve the effect that a single atom cannot achieve. Yang et al.^[^ ^33^ ^]^ found that the codoping of Mn–Se and Mn–Te resulted in a single‐layer WSe_2_ showing magnetic semimetallic properties. Although the pure monolayer WSe_2_ is a nonmagnetic semiconductor, the codoped systems have magnetic properties due to the introduction of an unpaired electron. Singh et al.^[^ ^34^ ^]^ studied the conditions under which Mn‐doped monolayers MoS_2_ behave ferromagnetically. The researchers found that undoped MoS_2_ exhibits diamagnetism at room temperature, with weak ferromagnetism, but paramagnetism at 100 to 300 K. When doped, only when there is a suitable distance between Mn and Mn can ferromagnetism appear, and as the doping concentration of Mn increases in MoS_2_, ferromagnetism dominates.

*Ni Doping*: Ni, also a transition metal, is itself ferromagnetic. Doping Ni makes the 2D transition metal sulfide also have good magnetic properties at room temperature, which provides a good foundation for magnetic devices working at room temperature. Gil et al.^[^ ^35^ ^]^ studied the magnetism of WSe_2_ after Ni doping, and also systematically studied the doped WSe_2_ using density functional theory. According to the calculations, Ni can only create low spin configuration antiferromagnetism at ambient temperature. Because of the hybridization of the d orbitals between the neighboring Ni atoms that close the gap in the spin‐up and spin‐down directions. The two neighboring Ni spins are coupled in opposite directions via a Se atom connecting the impurity pair. Such a mechanism can be attributed to the bond angle of Ni–Se–Ni, which is calculated to be 112° after the atomic relaxation. Thus, based on the Anderson–Goodenough–Kanamori rule, the preferred magnetic alignment for the two neighboring Ni atoms is antiferromagnetic. Olivas et al.^[^ ^31^ ^]^ studied the magnetism of Ni‐doped WS_2_. It can be observed from the M–H curve that the coercivity (*H*_c_) of the Ni‐doped WS_2_ increases with the extension of the reduction time, showing soft ferromagnetism (Figure 4e). The morphological defects caused by the morphological differences observed by HRTEM and the anisotropy caused by the interaction between particles also affect the magnetism. Martinez et al.^[^ ^36^ ^]^ prepared transition metal‐doped MoS_2_ nanocarbon nanotubes and studied their magnetism. At a temperature of 10 K, the magnetization has no hysteresis characteristics with the change of magnetic field, showing antiferromagnetism. As the temperature further increases, a hysteresis behavior with a coercivity of 175 Oe appears, indicating that it has room temperature ferromagnetism. The researchers speculate that its ferromagnetism is the result of the interaction between the active spin centers. The isothermal magnetization measurement of Ni‐doped MoS_2_ carbon nanotubes shows that it has strong room‐temperature ferromagnetism.

*Cr Doping*: In general, Cr doping is a useful technique for adjusting the magnetic of numerous systems. Similarly, the researchers think it is possible to investigate this method on 2D transition metal sulfides. Several investigations have repeatedly doped 2D transition metal sulfide systems, after which their magnetic alterations have been noticed. Zhang et al.^[^ ^37^ ^]^ studied the ferromagnetism of MoS_2_ after Cr doping, and according to the first‐principles calculation, it is shown that Cr induces spin polarization by substituting the S site, thereby generating magnetism. The original undoped MoS_2_ is known to be nonmagnetic, but when a Cr atom fills an S site, a significant DOS asymmetry is found near the Fermi level, resulting in a spin‐polarized state. Although doping has a limited effect on the high‐frequency permeability of MoS_2_, it has a significant effect on the dielectric properties and impedance matching of MoS_2_ nanosheets, making Cr doped MoS_2_ a potential electromagnetic microwave absorbing material. However, Yang et al.^[^ ^38^ ^]^ studied the magnetic properties of the Cr doped monolayer WS_2_, and found that the Cr doping does not show magnetism. The spin‐up and spin state densities are completely symmetric, and there is no spin splitting near the Fermi surface, so the intrinsic WS_2_ does not display magnetism externally. At the same time, the effect of strain on the magnetism of Cr‐doped WS_2_ monolayer was studied. The magnetic moment of Cr‐doped WS_2_ monolayer remains at 0 μ_B_ regardless of the tensile strain. In addition, Yang et al.^[^ ^32^ ^]^ found that Cr‐doped layered Td‐WTe_2_ has very large magnetoresistance and exhibits half‐metallic behavior. As shown in Figure 4f, the high crystal of pure Td‐WTe_2_ has a diamagnetism. Due to the existence of various defects, negligible weak ferromagnetism is generated under the inherent diamagnetic background, resulting in a step in the linear negative slope diamagnetic curve near the zero magnetic field. By introducing the Cr element into the layered Td‐WTe_2_, a stable tunable near‐room‐temperature ferromagnetism with *T*_c_ up to 283 K was achieved. In particular, the most important concern is the ability of Cr to induce and regulate the ferromagnetic order in layered Td‐WTe_2_. Figure 4g shows the zero field cooling and field cooling M–T curves of pure WTe_2_ under a 0.1 T magnetic field. Since the 0.1 T magnetic field is only the saturation field of defect‐induced ferromagnetism in WTe_2_, the moment of the M–T curve of diamagnetic pure WTe_2_ is positive rather than negative.

*V Doping*: According to the good performance of similar transition metals Mn, Cr, and other elements in doping, the researchers studied the magnetism and stability of the same transition metal V atom doped with different 2D transition metal sulfides and obtained some unique characteristics of V atom doping. Mekonnen and Singh^[^ ^39^ ^]^ studied the magnetism of V‐doped monolayer (ML) and bilayer (BL) MoS_2_. The results show that the substitution of V atoms at the Mo site is beneficial in energy, so the magnetic interaction of ML and BL MoS_2_ doped is obtained, showing from ferromagnetic oscillation to antiferromagnetic. The interlayer interaction in BL MoS_2_ will affect the magnetic properties after V doping. At the same time, for ML MoS_2_, because the DOS of the upper and lower spin channels is symmetrical, it shows that it has nonmagnetic properties. Jimenez et al.^[^ ^40^ ^]^ used the principle of magnetic LC resonance to detect the permeability of the V‐doped WS_2_ monolayer under illumination, and found that the magnetization of the V‐WS_2_ monolayer changed significantly. Guided by density functional theory calculations, this phenomenon was attributed to the carriers trapped in the magnetically doped state, which caused the magnetization of the V‐WS_2_ monolayer. The study also additionally found that the permeability of the monolayer depends on the laser intensity, confirming the optical control of the room temperature magnetic properties of the material. These findings provide a unique avenue for the development of RT optically controlled ferromagnetism and may establish new subfield‐optical spintronics. Duong et al.^[^ ^41^ ^]^ used spin‐polarized density functional theory to calculate the long‐range ferromagnetic ordering of V‐doped monolayer WSe_2_. It is found that V doping results in a fully occupied state in the valence band, which is inherent in the spin–orbit coupling, resulting in the presence of free holes in the valence band. This finding paves the way for the future preparation of carrier‐mediated room temperature 2D ferromagnetic semiconductors using magnetic dopants.

*Codoping of V, Nb, and Ta*: To improve some defects caused by single‐atom doping of 2D transition metal sulfides, a large number of researches focus on the codoping of various metal elements. The study found that among the methods of using various metal element doping systems to make ferromagnetic, the most form is V, Nb, and Ta three elements doped together, where the V doping system is metallic, and Nb and Ta doping system is semimetallic. Li et al.^[^ ^42^ ^]^ found that V, Nb, and Ta doped WS_2_ monolayer has ferromagnetism by first‐principles study. The ferromagnetism mainly comes from the coupling between the unpaired d orbital of the doped atomic and the adjacent W 5d and S 3p states. The first‐principles calculations of the doped samples show that the doped system maintains the geometric shape of the original WS_2_ monolayer, despite slight lattice distortion. Gao et al.^[^ ^43^ ^]^ used density functional theory to explore the reasons for the ferromagnetism of MoS_2_ by introducing holes in the narrow d‐band at the top of the Mo valence band. The hole states introduced by V, Nb, and Ta doping are ferromagnetically coupled, in which case the magnetic moment is suppressed by the spin singlet formation. When considering the spin–orbit coupling, for the MoS_2_ monolayer doped with V, Nb, and Ta, the Curie temperature is a function of the doping concentration. It is found that the maximum *T*_c_ is ≈170 K when the doping concentration is ≈9%. When the doping concentration is high enough, the magnetism is suppressed when the bandwidth exceeds the critical value. Experiments show that the shallow impurities in the MX_2_ monolayer are weakly bound, but have long‐range interaction to achieve room‐temperature ferromagnetism.

*Co Doping*: In general, it is common to introduce magnetism by doping with magnetic elements, and weak ferromagnetism can be observed in most cases. The researchers doped Co elements in 2D transition metal sulfides, which can be doped into single crystals with high efficiency to obtain excellent diluted magnetic semiconductors. Yang et al.^[^ ^44^ ^]^ studied the effect of Co‐X_6_ (X = S, N, O, and F) doping on the magnetic properties of monolayer WS_2_ by the first‐principles method. All monolayer WS_2_ alloys doped with Co‐X_6_ become magnetic and the pristine monolayer WS_2_ alloy was nonmagnetic since there was no spin density on any atoms which led to the TMM of 0 µ_B_. In addition, all the cluster Co‐X_6_ (X = S, N, O, and F) doped monolayer ZS_2_ (Z = Mo and W) alloys became magnetic due to the cluster dopants. The TMM was mainly contributed by the doped Co atoms for all the alloys. The magnetic coupling (ferromagnetic (FM) coupling or antiferromagnetic coupling) between Co and S atoms, and between S and W atoms were very complex for the doped alloys. Fan et al.^[^ ^45^ ^]^ also studied the magnetism of MoS_2_ doped with low and high concentrations of Co by first‐principles calculations. The calculation results show that monolayer MoS_2_ doped with Co element can obtain excellent diluted magnetic semiconductors at lower impurity concentrations. By studying different separation configurations, it is found that the doped atoms mostly produce ferromagnetic coupling in the nearest neighbor configuration. Additionally, we have found that the doped Co atoms prefer to stay in the nearest neighboring positions at high concentrations and couple with each other ferromagnetically. For Co doping, the induced spins on the nearby host atoms are parallel to that of the impurities. It indicates that the local structures around the impurities are deformed from the original prismatic configurations for Co doping at high impurity concentration although Co doping induces strong ferromagnetism into the doped system. Ahmed et al.^[^ ^46^ ^]^ studied the magnetic properties of WSe_2_ by Nb and Co codoping. Magnetic measurements were performed after physical ion implantation of 4 at% Co and different concentrations of Nb codoped WSe_2_ single crystals. The results show that both undoped and 4 at% Co‐doped WSe_2_ exhibit weak ferromagnetism, while Co and Nb codoped WSe_2_ have significantly enhanced magnetization. There are some defects in the original WSe_2_ single crystal, so the ferromagnetism is weak. Its magnetization is comparable to that of the ferromagnet, which may be related to the doping and defects of Co and Nb.

**Figure 5:**
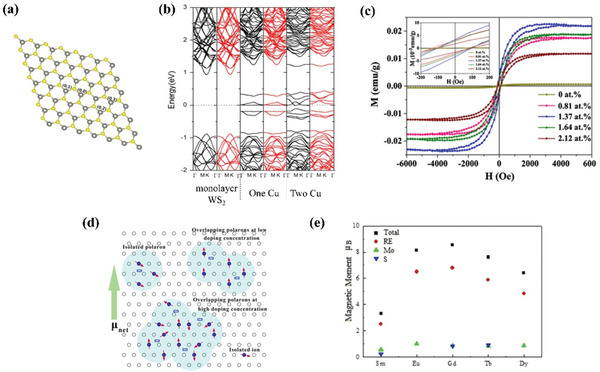
a) Schematic diagram of double Cu doping sites (yellow represents S atom, gray represents W atom). b) Band structure diagram of single‐layer WS_2_, single‐Cu‐doped WS_2_, and double‐Cu‐doped WS_2_. Reproduced with permission.^[^ ^50^ ^]^ Copyright 2015, Elsevier. c) Hysteresis curves of Dy‐MoS_2_. e) The schematic diagrams of Dy‐MoS_2_ magnet poles with different doping concentrations. Reproduced with permission.^[^ ^51^ ^]^ Copyright 2019, Elsevier. d) Calculated results of the total and local magnetic moments of rare earth (Sm, Eu, Gd, Tb, and Dy), molybdenum, and sulfur atoms in the rare‐earth‐doped MoS monolayer. Reproduced with permission.^[^ ^52^ ^]^ Copyright 2016, American Institute of Physics.

*Cu Doping*: In addition to the common doping of Mn, Fe, Co, etc., the magnetic regulation of Cu‐doped TMDs has gradually entered people's field of vision in recent years. Like Mn, Cu is not magnetic in its bulk phase, but studies have shown that introducing it into TMDs by doping can effectively induce its magnetism. Xia et al.^[^ ^47^ ^]^ prepared Cu‐MoS_2_ nanosheets and studied the magnetic properties. The results show that Cu doping successfully introduces strong ferromagnetism into MoS_2_ nanosheets. Zhang et al.^[^ ^48^ ^]^ studied Cu‐WSe_2_ with different doping concentrations and the effect of external strain on Cu‐WSe_2_ by first‐principles calculations. The results show that Cu doping can effectively introduce magnetism into WSe_2_. At low doping concentration, the magnetic moment is 5.0 µ_B_, while its bandgap is greatly reduced. Regarding the question of how the nonmagnetic Cu element in the bulk phase induces the magnetic properties of TMDs, the study shows that it may be due to the strong hybridization between the Cu 3d state and the adjacent atoms, resulting in the generation of spin‐split impurity bands, increasing the magnetic moment, thereby introducing magnetism.^[^ ^47^, ^49^ ^]^ In addition to single Cu atom doping, the researchers proposed some new ways to introduce Cu atoms to solve the problem that Cu doping cannot control the magnetic properties of some TMDs due to the influence of Jahn‐Teller distortion. Zhao et al.^[^ ^50^ ^]^ used density functional theory to study the effect of different concentrations of Cu‐doped WS_2_ on the magnetic properties, and proposed the concept of adjacent double copper doping. Calculations show that when WS_2_ is doped with only one Cu atom, it still exhibits nonmagnetic properties, while when doped with two adjacent Cu atoms, it exhibits ferromagnetic properties. Its doping position is shown in **Figure** **5**a, and the energy band structure diagram is shown in Figure 5b.

#### 4.2.2 Rare Earth Doping (Dy and Re)

In addition to the modification ways to replace transition metal atoms (TM atoms), rare earth element doping has also been gradually studied. Rare earth elements are composed of 15 kinds of lanthanides, scandium, and yttrium, which are mainly expressed as +3 valence. The electronic structure of rare earth elements is [Xe]6s^2^4f*^n^* (*n* = 0–14).^[^ ^53^ ^]^ Due to its great difference in the arrangement of 4f electrons, rare earth elements have abundant energy levels, and optical properties are very different from those of transition metal ions, it is necessary to study the effect of rare earth element doping on the magnetic properties of TMDs.^[^ ^54^ ^]^ Studies have shown that rare earth dopants possess unfilled 4f and 5d energy states and abundant electronic energy levels, which can provide strong spin–orbit coupling to tune the semiconducting properties of TMDs materials.^[^ ^55^ ^]^ Zhao et al.^[^ ^51^ ^]^ synthesized Dy‐MoS_2_ for the first time, and the synthesized samples successfully exhibited room‐temperature ferromagnetism (Figure 5c). The saturation magnetization value of Dy‐MoS_2_ can be regulated by different Dy doping concentrations. The schematic diagram of the magnetization poles with different doping concentrations is shown in Figure 5e. Theoretical analysis shows that ferromagnetism arises from the strong interaction between Mo3d, S4p, and Dy5d orbitals. Majid et al.^[^ ^52^ ^]^ studied the magnetic moment value and changing trend of MoS_2_ doped with various rare earth elements (Sm, Eu, Gd, Tb, and Dy) (Figure 5d), providing a theory for rare earth doping modification.

#### 4.2.3 Noble Metal Doping (Ag, Pd, Pt)

**Figure 6:**
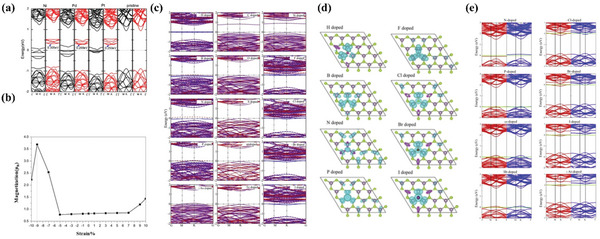
a) Band structure diagram of X‐WS_2_ (X = Ni, Pd, Pt). Reproduced with permission.^[^ ^56^ ^]^ Copyright 2016, Elsevier. b) Change of magnetic moment of Ag‐WS_2_ corresponding to strain change. Reproduced with permission.^[^ ^57^ ^]^ Copyright 2016, Elsevier. c) The band structure diagrams of pristine MoSe_2_ and MoSe_2_ doped with nonmetallic elements. d) The spin density of MoSe_2_ doped with different elements. Reproduced with permission.^[^ ^58^ ^]^ Copyright 2018, Elsevier. e) The spin‐polarized band structures of WS_2_ doped with different elements. Reproduced with permission.^[^ ^59^ ^]^ Copyright 2017, Elsevier.

Computational studies have shown that noble metal doping can also induce magnetism in nonmagnetic TMDs. Zhao et al.^[^ ^56^ ^]^ simulated the effects of doping with different noble metal elements (Ni, Pd, Pt) on the magnetic properties of WS_2_. The results show that the monolayer WS_2_ doped with Ni, Pd, and Pt all exhibit ferromagnetism. The magnetic moments generated by Ni‐WS_2_, Pd‐WS_2_, and Pt‐WS_2_ are 3.213 µ_B_, 2.841 µ_B_, and 2.920 µ_B_, respectively (**Figure** **6**a). Compared with other atoms, the Ni‐doped system has the lowest formation energy and the widest half‐metal gap. In addition to simply doping noble metals to control the magnetic properties, Chen et al.^[^ ^57^ ^]^ found that the magnetic moment of Ag‐doped monolayer WS_2_ is very easy to change with strain (Figure 6b). This is because the electronic and magnetic changes of Ag‐WS_2_ are determined by the strength of Ag—S and W—S bonds. Ag‐WS_2_ exhibits different properties of semimetal or metal with the change of strain, which provides a new idea for application in spintronic devices. Although in the above studies, noble metal doping shows the characteristics of inducing magnetism, the number of related studies is small, and they only stay in the calculation stage, and the types of TMDs covered are relatively simple. The effect of noble metal doping on the magnetic properties of TMDs needs to be further studied.

#### 4.2.4 Nonmetallic Elements Doping

To changing the chemical composition of TMDs by doping metal atoms, thereby changing the magnetic properties, the doping of nonmetal atoms has also been studied, and the magnetic properties have been successfully induced. Zhao et al.^[^ ^58^ ^]^ investigated the magnetic effects of doping with various nonmetallic elements (H, B, C, Si, N, P, As, O, S, Te, F, Cl, Br, I) on monolayer MoSe_2_ by first‐principles calculations. The results show that whether doping can induce magnetism depends on the parity of valence electrons and doping with an odd number of valence electrons can induce magnetism; conversely, doping with an even number of valence electrons cannot induce magnetism (Figure 6c). Meanwhile, MoSe_2_ doped with H, B, F, Cl, Br, and I elements have semimetallic properties due to 100% spin polarization around the Fermi level. Figure 6d shows the spin density of MoSe_2_ doped with different elements, and the results show that the spin density is mainly concentrated on the dopant atoms and Mo atoms, while the contribution of Se atoms is negligible. However, due to the influence of the thermodynamic driving force, the doped atoms can easily form clusters, making single‐atom doping unstable.^[^ ^60^ ^]^ Therefore, some researchers choose more advantageous cluster doping to control the magnetism of TMDs. Yang et al.^[^ ^44^ ^]^ used a first‐principles approach to study the effect of cluster Co‐X_6_ (X = S, N, O, and F) doping on the magnetic properties of monolayer WS_2_. Studies have shown that all cluster‐doped monolayers of WS_2_ are magnetic and can be used in spintronic devices. At the same time, some studies have shown that the codoping of metal atoms and nonmetal atoms can also well control the magnetic properties of TMDs.

#### 4.2.5 p‐ and n‐Type Doping

In addition to discussing the effects of specific doping atom species on the magnetic properties of TMDs, some researchers have also studied the effects of p‐type and n‐type doping on the magnetic properties. p‐type and n‐type doping refers to a modification means for the intrinsic TMDs semiconductors to be transformed into n‐type or p‐type semiconductors after doping with appropriate elements at cation or chalcogenation sites. At present, extensive research has confirmed that p‐type and n‐type doping can effectively control the electronic structure and change the optoelectronic properties of semiconductors. With the deepening of research and the entry of spintronic devices into the field of vision of researchers, people realize that p‐type and n‐type doping can not only be used to control the degree of freedom of electrons, but also control the freedom of electron spins, to realize the control of magnetic properties. Mahmood et al.^[^ ^61^ ^]^ performed a half‐metallic ferromagnetic analysis of XBeO_3_ (X = Li, Na, K, Rb, and Cs) using a first‐principles approach. It is found that p‐type half‐metal ferromagnetism originates from a hole‐mediated double‐exchange mechanism, and the strong p–p coupling between p‐X and p‐O states of this mechanism favors half‐metal ferromagnetism. Wei and Zhang^[^ ^62^ ^]^ computationally investigated the electronic structures and magnetic properties of N‐doped ZnO, Cu‐doped ZnO, and Cu, N codoped ZnO. It is found that Cu, N codoped ZnO has a more obvious p‐type doping effect than N‐doped ZnO, and the codoped Cu and N can significantly increase the ferromagnetism of the system. The effects of p‐type and n‐type doping of TMDs on the magnetic properties have also been gradually studied. Zhu and Zhang^[^ ^59^ ^]^ systematically investigated the properties of p‐type (group VA elements N, P, As, or Sb) and n‐type (group VIIA elements Cl, Br, I, or At) doped monolayer WS_2_ systems using first‐principles calculations. It is found that the impurity positions brought by different doping types are different, and the impurity bands of p‐type and n‐type are located just above the VBM and just below the CBM of the original monolayer WS_2_ system, respectively (Figure 6e). The systems doped with n‐type elements all exhibit magnetic properties, the Cl and Br doped systems behave as magnetic metals, and the I and At doped systems behave as magnetic semimetals. In p‐type doping, N‐doped systems behave as magnetic semiconductors, while P, As, and Sb doped systems behave as nonmagnetic metals. Zhao et al.^[^ ^63^ ^]^ simulated the replacement of Se atoms in MoSe_2_ monolayers with V and VII atoms, the properties of n‐type (VIIA group elements Cl, Br, I) and p‐type (VA group elements N, P, As) doped MoSe_2_ monolayers were studied. The results show that except As doping, all other elements can induce magnetism, and the magnetism mainly originates from the p–d hybridization between the p orbital of the doped atom and the d orbital of three adjacent Mo atoms. The above studies show that the effects of p‐type and n‐type doping on the magnetic properties are of great significance and can also provide guidance for the magnetic regulation of TMDs materials. However, at present, p‐type and n‐type doping methods are more used in the modification of semiconductor materials for optical devices. There is still a lack of extensive and systematic research on the effects of p‐type and n‐type doping on the magnetic properties of materials.

## 5 Structure Regulation Strategy

### 5.1 Composites

**Figure 7:**
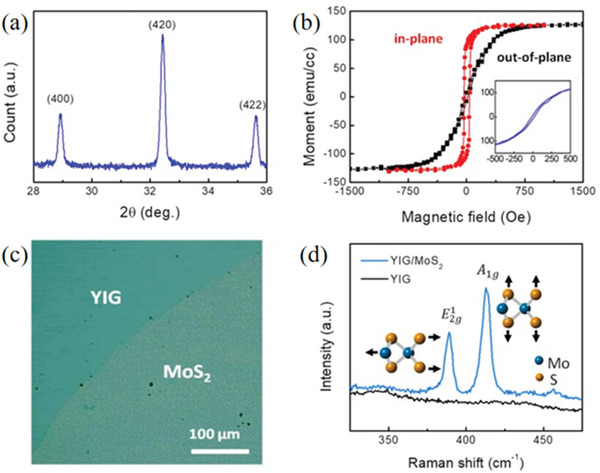
Single‐layer MoS_2_ transferred onto a YIG thin film. a) The XRD pattern of the annealed YIG. b) Hysteresis loops obtained along the perpendicular (hard‐axis, black curve) and in‐plane (easy‐axis, red curve) directions at 300 K. Inset: the center of the out‐of‐plane hysteresis curve exhibiting detectable coercivity, c) optical microscopic image of the MoS_2_ monolayer on YIG, and d) Raman spectra of YIG and the MoS_2_ single‐layer on YIG. Reproduced with permission.^[^ ^64^ ^]^ Copyright 2020, Wiley‐VCH.

At very low temperatures, the valence band of the semiconductor is full. After the semiconductor is thermally excited, some electrons in the valence band will cross the forbidden band into the higher energy empty band, so that the empty band will become the conduction band, and the electrons in the conduction band will fall into the holes, resulting in the “electron–hole pairs” disappears. The above process is called compounding. Research has shown that the energy released during compounding thus makes the material magnetic. Therefore, in addition to the common elemental doping and vacancy defects, transition metal sulfides can also obtain certain magnetic properties by compounding with different materials. In general, the composite system tends to have stronger adsorption properties than pure 2D transition metal sulfides, which makes it better for photocatalytic applications. It is further demonstrated that the catalytic performance of 2D transition metal elemental sulfide‐based photocatalysts can be effectively improved if they can be magnetized using magnetic fields. In conclusion, exploring the magnetic properties of 2D transition metal sulfide‐based composites is important for practical applications in both optoelectronic devices and spintronics. Tsai et al.^[^ ^64^ ^]^ prepared YIG/MoS_2_ composite, and showed that the composite possesses room‐temperature ferromagnetism that is not present in pristine pure MoS_2_ (**Figure** **7**a,b). It was further shown that the appearance of this magnetic feature was caused by the magnetic proximity effect of YIG and the antiferromagnetic coupling at the interface (Figure 7c,d) and the composite system effectively modulated the magnetic characteristics of MoS_2_. This study provides a layer‐selective approach to studying the magnetic interaction/configuration in the YIG/MoS_2_ bilayers.

**Figure 8:**
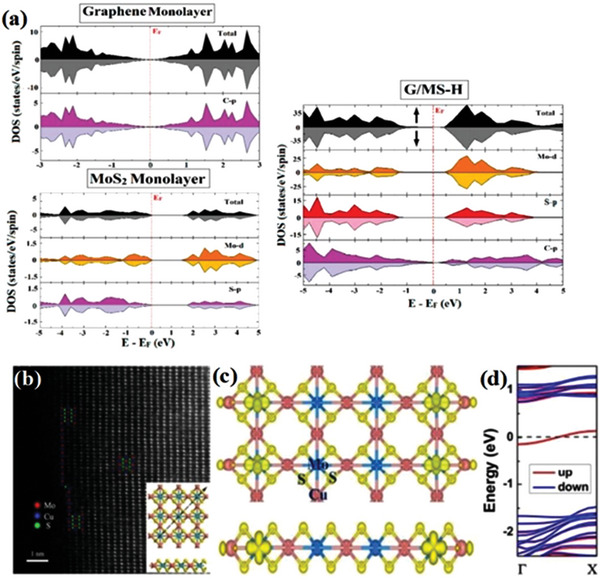
a) Calculated spin‐resolved total and partial DOS of pristine graphene monolayer, MoS_2_ monolayer, and G/MS‐H. Reproduced with permission.^[^ ^66^ ^]^ Copyright 2021, Elsevier Ltd. b) STEM image with Cu and Mo atoms at the edge in a Cu_2_MoS_4_ sheet. The inset figure shows the optimized geometry structure based on the STEM observation, c) spin charge density distribution of Cu_2_MoS_4_ sheet with metal edges, and d) the electronic band structure based on HSE06 calculations of Cu_2_MoS_4_ nanoribbon with Cu terminated edges. Reproduced with permission.^[^ ^67^ ^]^ Copyright 2016, Royal Society of Chemistry.

As research progressed, researchers have found that 2D transition metal sulfides could be compounded with graphene, thus serving to compensate for the lack of bandgap and ferromagnetic ordering in graphene proper, taking full advantage of graphene's high carrier mobility and enabling its practical application in spintronics. Wang et al.^[^ ^65^ ^]^ designed a novel composite of graphene and Mn‐doped monolayer MoS_2_. The magnetic and electronic structures of graphene/Mn‐doped monolayer MoS_2_ heterostructures were investigated by using density flooding theory (DFT) and van der Waals correlation (DFT‐D). It was found that the heterostructures showed larger magnetic moments and more stable FM states than isolated Mn‐doped MoS_2_ monolayers. These findings provide a new approach to facilitate the design of spintronic devices that require both stable ferromagnetism and a limited bandgap. Similarly, Singla et al.^[^ ^66^ ^]^ showed that the graphene/MoS_2_ heterostructure (G/MS‐H) has excellent electronic properties because it contains features of both graphene and MoS_2_, and it was shown by first‐principles calculations that this heterostructure has a small bandgap (40 meV) and zero magnetic moment in its original form, further demonstrating that after compounding it can greatly improve the magnetic properties of the material (**Figure** **8**a).

Zhang et al.^[^ ^67^ ^]^ found that the ferromagnetic behavior at room temperature could be observed in ternary layered Cu‐MoS_2_ nanoribbons where both coercivity and magnetization saturation increased with decreasing temperature. As shown in Figure 8b. The energy calculations show that the studied Cu_2_MoS_4_ nanoribbons have a ferromagnetic ground state and the spin charge distribution is shown in Figure 8c, and the Mo atoms at the edges have the main contribution to the ferromagnetism of the Cu_2_MoS_4_ nanoribbons (Figure 8d). This study also proves that transition metal sulfides obtain certain magnetic properties in combination with different materials and the magnetic properties’ magnitude is temperature dependent.

**Figure 9:**
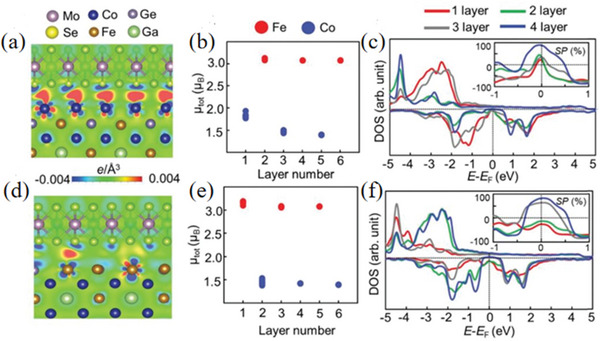
a,d) Atomic structure and interfacial electronic charge redistribution at the MoSe_2_/CFGG interface, b,e) layer‐resolved magnetic moments for Fe and Co atoms in CFGG, and c,f) layer‐resolved DOS of CFGG. The up and bottom rows correspond to the Co‐ or FGG‐termination, respectively. Insets in (c) and (f) show layer‐resolved spin polarization in CFGG. Reproduced with permission.^[^ ^68^ ^]^ Copyright 2022, Royal Society of Chemistry.

Larionov et al.^[^ ^68^ ^]^ found that a large number of predicted and synthesized 2D materials broadened the magnetic interface design capabilities for spintronics applications. The observed cohesion energies between MoSe_2_ and CFGG were equal to 51.1 and 29.3 meV Å^−2^ for Co and FGG terminals, respectively. The latter value is larger than that of the h‐BN/CFGG heterostructure, which is related to the stronger interfacial interaction observed from the electronic charge redistribution (**Figure** **9**). However, such interfacial interactions may also be associated with a combination of weak electrostatic and van der Waals forces, and the possibility of fine‐tuning the interfacial interactions between electrodes and spacer materials by diversifying structural and electronic properties to provide efficient spin transport suggests that interfacial interactions in composites can likewise enable magnetic energy changes, providing a 2D transition metal‐sulfide compound composite with a new pathway for magnetic modification of 2D transition metal‐sulfide composites.

**Figure 10:**
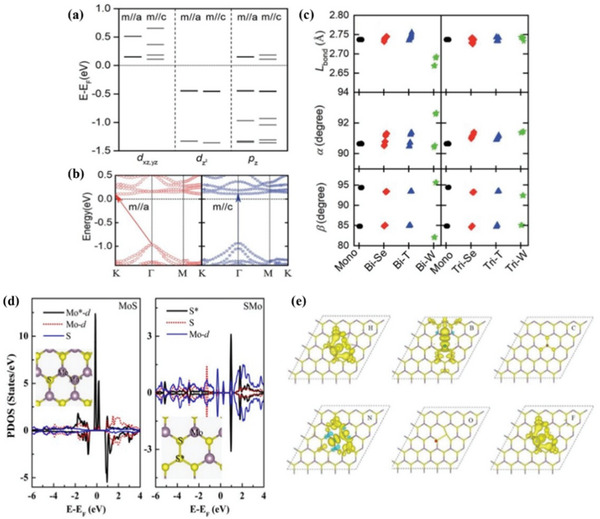
a) Effects of the spin axis on the wave function characteristics at G points for Cr‐d and Se‐p orbitals of CrI_3_/WSe_2_ heterostructures. b) Band structure of CrI_3_/WSe_2_ heterostructures for the magnetic moment along the in‐plane axis (left panel) out‐of‐plane axis (right panel). The arrows show that the bandgap is indirect with m//a and direct with m//c. c) Bond lengths (*L*_bond_) and bond angles for bilayer and trilayer CrI_3_/WSe_2_ heterostructures with three different stacking models. Reproduced with permission.^[^ ^69^ ^]^ Copyright 2019, Royal Society of Chemistry. d) PDOSs projected on Mo*‐d orbital, Mo‐d orbital, S atoms, and S* atom in MoS and SMo system. e) The magnetization density of the X‐SMo systems. The yellow and blue isosurfaces correspond to spin‐up and spin‐down charges, respectively. Reproduced with permission.^[^ ^70^ ^]^ Copyright 2019, Elsevier Ltd.

The electronic and magnetic properties of the van der Waals CrI_3_/WSe_2_ heterostructure were investigated by Ge et al.^[^ ^69^ ^]^ using first‐principles calculations. In addition to the application of in‐plane external magnetic fields, this study also demonstrated that the spin direction can be transferred from out‐of‐plane to in‐plane by stacking with Se_2_ layers. In particular, for most of the CrI_3_/WSe_2_ stacking configurations, the out‐of‐plane spin quantization axis of the monolayer CrI_3_ is tuned to the in‐plane. It was further revealed that the shift of susceptibility magnetization direction mainly originates from the hybridization between Cr‐d and Se‐p orbitals (**Figure** **10**). It is demonstrated that the electronic structure and magnetic anisotropy behavior of the heterostructures formed after compounding are altered and the magnetic orientation can be tuned, providing more ways to further improve the properties of the materials such as photocatalysis.

### 5.2 Metal and Nonmetal Adsorption

2D transition metal sulfides and semiconductor materials with similar 2D structures have attracted attention in electronics and optoelectronics due to their special structures and good catalytic properties. Meanwhile, the large surface area of 2D materials makes them extremely sensitive to different reactions and can also be widely used in the field of gas sensing and adsorption, while the adsorption of metallic elements can achieve the effect of surface modification thus greatly enhancing the magnetic properties. Further studies have shown that the adsorption of nonmetallic elements on the surface of 2D materials generates local magnetic moments, which provide a new direction for the introduction and regulation of magnetic properties of 2D transition metal sulfides.

Wang et al.^[^ ^71^ ^]^ investigated the adsorption of noble metals (including Pd, Pt, and Au atoms) on MoSe_2_ monolayers using density flooding theory calculations. The results of adsorption energies showed that all adsorbed MoSe_2_ monolayers of Pd, Pt, and Au are energy stable. Based on the energy band structure calculations, all noble metal adsorbed MoSe_2_ systems exhibit semiconductor properties.

In general, ferromagnetism is usually considered to be incompatible with superconductivity. Therefore, the coexistence of superconductivity and ferromagnetism is usually observed only in a few tuned multicomponent structures where the two competing electronic states come from different structural components. The induction of ferromagnetism was studied by Zhu et al.^[^ ^72^ ^]^ The induction of ferromagnetism in 2D superconducting NbSe_2_ was studied using surface molecular adsorption, and the surface showed that superconductivity and ferromagnetism can coexist in 2D nanomaterials. Surface‐structural modulation of the ultrathin superconducting NbSe_2_ by polar reductive hydrazine molecules triggers a slight elongation of the covalent Nb—Se bond, which weakens the covalent interaction and enhances the ionicity of the tetravalent Nb with unpaired electrons, yielding ferromagnetic ordering, and the surface showed that superconductivity and ferromagnetism can coexist in 2D nanomaterials.

Studies have shown that the performance of nanoelectronic devices is highly dependent on the morphology of the defects and the quality of the low‐dimensional material. Gao et al.^[^ ^70^ ^]^ reported the magnetic and electronic properties of MoS_2_ monolayers with two types of antilocalization defects obtained based on density generalization theory. In general, the adsorption of nonmetallic elements on metal oxides is more favorable than that of transition metal atoms (Figure 10d). For example, the binding energy of F atoms on metal oxides reaches 5.74 eV (Figure 10e), which is significantly greater than the corresponding binding energy of transition metal atoms on metal oxides. The adsorption of C atoms does not affect the magnetic properties of the substrate, while the adsorption of nonmetallic elements alters the magnetic properties of the MoS_2_ system, yielding nonmagnetic ground states of the O‐MoS and O‐SMo systems. It can be seen that the presence of conventional magnetic ions in 2D materials may not be a necessary condition for their magnetic properties, and the adsorption of nonmetallic elements on the surface of 2D materials also affects the magnetic properties. It can be expected that surface molecular adsorption will become a powerful tool to regulate the spin ordering of 2D materials in future research directions.

### 5.3 Phase Regulation

The magnetism of 2D transition metal sulfides can also originate from phase transitions, while the monolithic and monolayer structure phase transitions differ mainly in energy and are related to the van der Waals interactions between the layers. The most fundamental cause of the process of magnetic and magnetic energy change is the change in the relevant factors affecting the electron spin, which is indirectly reflected in the increase of the migration potential barrier.

**Figure 11:**
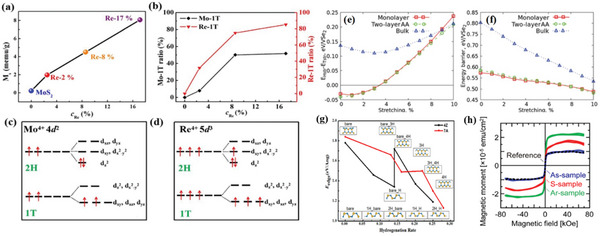
a) Re concentration dependent saturated magnetization (Ms) of all the samples. b) 1T structural ratios of Mo and Re atoms versus Re concentration. Electron orbital degenerate states in 1T and 2H structures of c) Mo atoms and d) Re atoms are shown. Reproduced with permission.^[^ ^73^ ^]^ Copyright 2018, AIP. Energy difference between e) the H and T structures of VSe_2_ and f) the energy barrier as functions of stretching in (a) and (b) lattice directions. Lines of different colors correspond to systems with different numbers of layers. g) The edge formation energy (*E*_edge_) of WS_2_NRs varies with the edge hydrogenation rate. Illustrations represent WS_2_NR models with different edge hydrogenation modes, where their positions correspond to their *E*_edge_, respectively. h) Hysteresis curves of As‐sample (blue), S‐sample (red), Ar‐sample (green), and reference sample (black dashed line) at 300 K. Reproduced with permission.^[^ ^75^ ^]^ Copyright 2019, Royal Society of Chemistry.

Xia et al.^[^ ^73^ ^]^ synthesized Re‐doped MoS_2_ nanosheets by a simple hydrothermal reaction and annealing process. The doping of Re induced a 2H‐1T transition in the coordination structure of Re and Mo. Characterization showed (**Figure** **11**a,b) that the Re‐MoS_2_ material system exhibited room‐temperature ferromagnetism, and the magnetism was caused by an increase in the net magnetic moment of the Re atoms and the 1T‐coordinated Mo atoms (Figure 11c,d), indicating that phase modulation is one of the effective methods to regulate the magnetism. Xing et al.^[^ ^74^ ^]^ fabricated amorphous MoSe_2_ by hydrothermal method and adjusted the amorphous degree by the postannealing method. The amorphous MoSe_2_ has achieved a large room temperature ferromagnetism, which diminishes during crystallization and phase transition from 1T to 2H structure. Theoretical calculations show that the amorphous MoSe_2_ exhibits higher magnetization intensity compared to the crystalline 1T and 2H phases and that the magnetization intensity mainly comes from the d orbitals of the Mo atoms. The above study also provides supporting evidence for the room‐temperature ferromagnetism of amorphous TMDs, which opens a new avenue for the future application of MoS_2_ nanosheets in spintronic devices.

Pushkarev et al.^[^ ^75^ ^]^ performed first‐principles calculations of the magnetic and electronic properties between the two structural phases (H, T) of VSe_2_. The calculations proved that the transition between the two phases has a rather low energy barrier (0.60 eV for a single layer). And stretching beyond 3% leads to a phase transition from H to T in the ground state configuration of both monolayers and bilayers of VSe_2_ (Figure 11e). Another effect of stretching is the reduction of the energy barrier for migration between the different conformations (Figure 11f), thus demonstrating that stress induces phase transitions that lead to changes in the magnetic properties of the material and that the ferromagnetic conformation is the ground state of all systems with stable and intermediate atomic structures.

Wang et al.^[^ ^76^ ^]^ also investigated the effects of strain and stacking modes on the electronic structure, valley bottom polarization, and magnet crystal anisotropy of layered VTe_2_ by first‐principles calculations. The results show that the stacking mode can significantly affect the valley bottom polarization of layered VTe_2_. AB stacking mode preserves the spatial inversion symmetry, leading to a significant decrease or even disappearance of the valley bottom polarization. On the contrary, the AA stacking mode maintains a larger valley bottom polarization because the spatial inversion symmetry is broken. It can be concluded that different motion energies in different structural models and the appropriate temperatures between different structural phase transitions also affect the electronic structure and magnetic energy in the intermediate stages of structural phase transitions.

### 5.4 Size or Edge Regulation

Magnetism in 2D materials can be modulated by changing the edge state, such as by inducing defects (vacancies, dislocations, grain boundaries) or absorbing nonmetals such as H, O, C, B, N, and F,^[^ ^77^ ^]^ the ferromagnetism of the material changes. Induced defects such as vacancies can increase the unsaturated atoms at the edges, resulting in magnetism.^[^ ^78^ ^]^ Atoms such as H, O, and C are adsorbed to eliminate edge suspension bonds (excess unpaired electrons, known as suspension bonds). Because of the existence of suspended edge bonds, the edge activity is very high, and the edge is passivated by adsorption of other atoms, thus making the performance more stable.^[^ ^79^ ^]^ In addition, the edge length and layer thickness of the 2D material will also affect its ferromagnetism.^[^ ^78^, ^80^ ^]^

Due to their different edge structures or sizes, 2D materials exhibit a variety of magnetic properties. The stability and ferromagnetism‐related physical properties of ferromagnetic 2D transition metal dihalides with different edge structures and sizes have been explored by many researchers. Ouyang et al.^[^ ^81^ ^]^ studied the structural stability, and electronic and magnetic properties of tungsten disulfide nanoribbons (WS_2_NRs) using first‐principles. The study found that when the edges are exposed, zig‐zag edge WS_2_NRs (ZWS_2_NRs) and armchair edge WS_2_NRs (AWS_2_NRs) are ferromagnetic metals and nonmagnetic semiconductors, respectively. After edge hydrogenation, WS_2_NRs exhibit different structural stability and electronic structure according to the edge hydrogenation mode (Figure 11g). Hydrogenated ZWS_2_NR remains ferromagnetic and metallic, while AWS_2_NR changes from nonmagnetic to magnetic when at least one edge is partially hydrogenated. Wang et al.^[^ ^79^ ^]^ investigated the magnetic properties and stability of ZMoS_2_NRs after passivating them with O‐atoms and based on first‐nature principle calculations. With the computational results, the authors concluded that O‐passivation not only effectively modulates the magnetic properties of ZMoS_2_NRs and induces local ferromagnetic states, but also greatly improves the stability of ZMoS_2_NRs because the edges terminated by O atoms can eliminate edge dangling bonds.

Ahmed et al.^[^ ^82^ ^]^ demonstrated that the annealing of MoS_2_ nanoparticles at different temperatures can induce ferromagnetism in pure MoS_2_ to induce ferromagnetism without doping with any foreign elements. However, the unannealed MoS_2_ exhibited antimagnetic behavior. The magnetization strength initially increases at annealing temperatures of 600 and 700 °C, then decreases at 800 °C and increases sharply at 1000 °C. The results indicate that the concentration of sulfur vacancies and edge‐end structures in MoS_2_ is strongly dependent on annealing temperature, which is a key factor in promoting ferromagnetism in annealed MoS_2_. Thus, this work could provide a basis for understanding the mechanism of magnetism caused by defects or edge structures and for developing a high‐quality diluted magnetic semiconductor based on 2D materials. Shirokura et al.^[^ ^83^ ^]^ measured the hysteresis curves of sputtered MoS_2_ deposition samples and their annealed samples in S vapor and Ar. The relationship between crystallinity and edge‐induced ferromagnetism was studied. It can be clearly observed from their hysteresis curves (Figure 11h) that the saturation magnetization values of the S and Ar annealed samples are greater than those of the directly deposited samples. Combined with other characterization methods, the author concludes that the periodicity of the grain and the shape of the grain boundary are important factors for edge‐induced ferromagnetism in the MoS_2_ film.

The above study shows that the edge state has a great influence on the magnetic properties of TMDs, so the method of regulating the magnetic properties by changing the edge state is of research significance and can also provide guidance for the magnetic regulation of TMDs materials.

## 6 External Field Treatment

### 6.1 Lattice Strain

Strain engineering is an effective method to modulate the magnetic properties of 2D materials to expand their applications in spintronics.^[^ ^84^ ^]^ Strain is well suited to the requirements of modulating the bandgap and inducing spin polarization without introducing impurity atoms. It plays an important role in designing the electronic structure and magnetism of 2D materials by varying bond lengths, bond angles, and electron–phonon coupling effects.^[^ ^85^ ^]^ Therefore, the ferromagnetism of 2D materials mediated by strain engineering has been extensively investigated.

**Figure 12:**
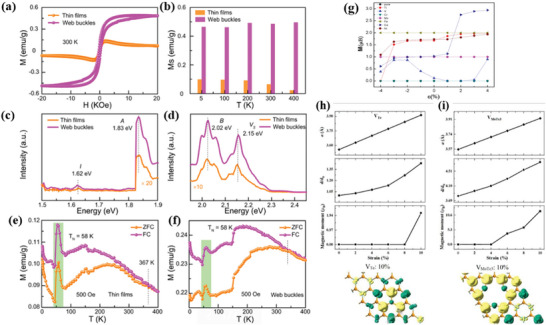
Ferromagnetic effect of MoS_2_ sheet and mesh. a) The M–H curves of MoS_2_ sheets and buckles at 300 K, b) Ms‐T of MoS_2_ flakes and mesh buckles, c) FL excitation spectra, and d) FL emission spectra at 1.83 eV excitation. e,f) M–T curves of MoS_2_ flakes and buckles. Reproduced with permission.^[^ ^87^ ^]^ Copyright 2020, American Institute of Physics. g) Relationship between magnetic moment and strain of TM doped MoTe_2_ monolayer. Reproduced with permission.^[^ ^7^ ^]^ Copyright 2017, Springer US. h) V_Te_ and i) V_MoTe3_ vacancy defects. The calculated magnetic moment, strain parameters (d/d0), lattice parameters, and spin density distribution under 10 % tensile strain (yellow isosurface describes the distribution of positive spin density). Reproduced with permission.^[^ ^23^ ^]^ Copyright 2021, Elsevier.

In recent years, several relationships between strain and related magnetic property modulation have been derived from the study of pure compressive‐tensile strain, strain and material doping, and strain and defects. Kahnouji et al.^[^ ^86^ ^]^ performed density functional calculations and Monte Carlo simulations to investigate the structural, electronic, magnetic, and thermodynamic properties of Co/WS_2_ nanospheres. Calculations of the electrical and magnetic properties of this system under various compressive and tensile strains show that a tensile strain of ≈4% can effectively improve the thermal stability of semimetallic ferromagnetism in Co/WS_2_ nanolayers. Ren et al.^[^ ^87^ ^]^ prepared MoS_2_ films by polymer‐assisted deposition and observed strain‐induced ferromagnetism in curved molybdenum disulfide films (**Figure** **12**a–f). After buckling, the saturation magnetization (Ms) at 300 K (0.486 emu g^−1^) is 7.5 times higher than that of the planar film (0.065 emu g^−1^), while the linear temperature coefficient (*χT*) of the E^1^_2g_ mode bent molybdenum disulfide film is reduced to one third. The results show that biaxial tensile strain plays an important role in magnetic modulation, which provides a feasible way for the manufacture and research of strain‐dependent spintronic devices.

Liu et al.^[^ ^7^ ^]^ used first‐principles calculations to study the electronic and magnetic properties and strain effects of MoTe_2_ monolayers doped with transition metal (TM) Ti, V, Cr, Mn, Fe, Co, and Ni atoms (Figure 12g). It can be observed that the magnetic moment of Ti, Co, and Ni doped MoTe_2_ sheets increases with increasing of strain, while the magnetic moment of V doped MoTe_2_ sheets oscillates with the increase of strain. The authors attribute this to the fact that the elastic strain applied to the TM‐doped MoTe_2_ monolayer system leads to the redistribution of electrons in the TM‐3d state, resulting in a magnetic state transition in the doped system.

Kanoun et al.^[^ ^23^ ^]^ studied the effects of vacancy defects and biaxial strain on the structure, electronic and magnetic properties of monolayer MoTe_2_ by first‐principles calculations. They found that the VTe and VMoTe_3_ vacancy configurations are nonmagnetic, but when they apply a biaxial tensile strain, the nonmagnetic defect system shows a magnetic state (Figure 12h,i). They attribute this change to the relative change of atomic spatial position, which leads to further delocalization of electrons and changes in the bonding effect around vacancies. They conclude that vacancy defects and strain can alter the electronic and magnetic properties of monolayer MoTe_2_, making it a potential candidate for spintronic applications. Numerous studies have shown that the introduction of strain can induce and control magnetism in some 2D TMDC. Therefore, the strain method has also become a common method and has long been used to control the electrons and magnetism of nanostructures, which will provide a new way for spintronics and strain electronics.

### 6.2 Electronic or Plasma Treatment

**Figure 13:**
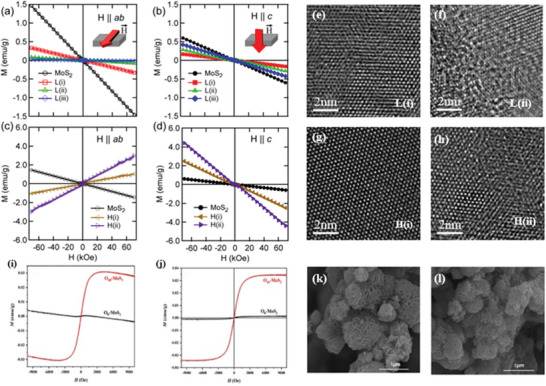
Changes of magnetization curves of original molybdenum disulfide and electron beam irradiated samples at 300 K along in‐plane, a,c) and out‐of‐plane, and b,d) directions. e–i) HRTEM images of electron beam‐irradiated samples. Reproduced with permission.^[^ ^8^ ^]^ Copyright 2016, American Institute of Physics. M–H curves of O_0_‐MoS_2_ and O_40_‐MoS_2_: i) before subtracting the linear part of the high field, j) after subtracting. SEM images of k) O_0_‐MoS_2_ and l) O_40_‐MoS_2_. Reproduced with permission.^[^ ^9^ ^]^ Copyright 2022, Springer US.

In addition to the mainstream methods to enhance the ferromagnetism of 2D transition metal dichalcogenides, it can also be achieved by electron irradiation induction, O plasma treatment, external electric field, and electron (nonhole) injection. Han et al.^[^ ^8^ ^]^ studied the electron irradiation‐induced magnetic phase transitions and defects in layered MoS_2_ single crystals by changing the electron dose and acceleration energy. Through the experimental results, they believe that this simple electron irradiation is an effective method to control the magnetism of single or several layers of TMDs in spintronics and quantum information devices. The magnetization curves of the original molybdenum disulfide and electron beam irradiated samples at 300 K change along the in‐plane and out‐of‐plane directions, respectively, as shown in **Figure** **13**a–d. At the same time, the HRTEM images before and after irradiation also show that the increase of various defects after irradiation is the main reason leading to the change of magnetic properties (Figure 13e–h). Therefore, magnetic induction by electron irradiation, which is simpler than chemical methods and can effectively induce large magnetic moments, is now being favored by more and more researchers.

In addition to obtaining dilute magnetic semiconductors with distinct ferromagnetic properties by introducing magnetic ions to occupy lattice sites with ferromagnetic exchange interactions in the semiconductor, nonmagnetic ion doping can also induce ferromagnetism. Xie et al.^[^ ^9^ ^]^ prepared nanoflower‐like MoS_2_ by hydrothermal method. Through some characterization methods, it is proved that the structure of MoS_2_ changes after O plasma treatment. At the same time, the magnetization measurement at 300 K shows that the ferromagnetism in the prepared MoS_2_ is weak, and its saturated ferromagnetism is 0.0012 emu g^−1^. However, after O plasma treatment, the weak ferromagnetism is strongly enhanced, and the saturation magnetization is 0.0343 emu g^−1^. The M–H curves of molybdenum O_0_‐MoS_2_ and O_40_‐MoS_2_ as shown in Figure 13i,j, and the first‐principles calculations show that the possible origin of ferromagnetism is due to the enhancement of ferromagnetism by O. Moreover, the morphologies of O_0_‐MoS_2_ and O_40_‐MoS_2_ were studied by scanning electron microscopy, and the obtained images were shown in Figure 13k,l. As shown in the figure, the morphology of O‐MoS_2_ did not change after O plasma treatment. Therefore, O plasma treatment will not lead to structural collapse. The introduction of nonmagnetic ions such as B, N, and F has also been studied by some scholars and induced strong room temperature ferromagnetism through the appropriate process.^[^ ^88^ ^]^

The regulation of ferromagnetism can also be achieved by injecting charge (electrons and holes). Li et al.^[^ ^89^ ^]^ used the first‐principles calculations to find that both ferromagnetism and valley polarization in the MoTe_2_ monolayer can be induced by charge injection. They believe that the reason for this phenomenon is due to the exchange splitting at the top of the valence band and the bottom of the conduction band. The origin of ferromagnetism is explained by the Stoner criterion.

Through the above methods, the ferromagnetism of TMD materials can be regulated, which can be more intuitively observed by the summary of the representative TMDs magnetic performance in **Table** **1**. However, the development of spintronic devices is still in the basic stage, the related regulation strategies both have advantages and disadvantages to enhance the ferromagnetic of 2D TMDs materials (summarized in **Table** **2**). It is expected to combine diluted magnetic semiconductors, antiferromagnetic semiconductors, and superconductors to form new heterojunctions and explore more new electronic devices. Therefore, there are still many challenges and endless opportunities in the research of magnetic properties of TMD materials.

**Table 1:** Summary of the representative TMDs magnetic performance

Strategies	Methods	Materials	Curie temperature [K]	Magnetic moment [µ_B_ emu g^−1^]		Coercive field [Oe]	Conducting type	Magnetic state	Refs.
				Maximum value	Room‐temperature value				
Vacancy introduction	Mo vacancy	Mo vacancy MoSe_2_	–	3.27 µ_B_	–	–	Semiconductor	Long‐range antiferromagnetic	[22]
		Mo vacancy MoTe_2_	–	2.53 µ_B_	–	–	Semiconductor	Magnetic ordering	[23]
	S vacancy	S vacancy 1T@2H‐MoS_2_	395 K	0.25 µ_B_/Mo	–	–	Semiconductor	Room‐temperature ferromagnetism	[24]
		S vacancy Mn‐doped monolayer MoS_2_	–	1.516 µ_B_	–	–	Semiconductor	Ferromagnetism	[25]
Element doping	Cu doping	Cu‐doped MoS_2_ nanosheets	930 K	–	0.023 emu g^−1^	–	Semiconductor	Long‐range ferromagnetism	[47]
		Cu‐doped SL‐WSe_2_	–	5.0 µ_B_	–	–	Semiconductor	Ferromagnetism	[48]
		Cu‐doped WS_2_ monolayer	–	0.864 µ_B_	–	–	Semiconductor	Ferromagnetism	[50]
		Cu‐doped Cu_2_MoS_4_ nanosheets	–	–	0.0346 emu g^−1^	–	Semiconductor	Room‐temperature ferromagnetic	[51]
	Cr doping	Cr‐doped MoS_2_	–	1.072 µ_B_	–	–	–	–	[37]
		Cr‐doped Td‐WTe2	–	–	4.20 emu g^−1^	–	1.24 eV	–	[32]
	Fe doping	Fe‐doped MoS_2_	–	–	–	143.7 Oe	–	–	[28]
		Fe‐doped WS_2_	–	–	–	–	0.066–1.243 eV	–	[31]
	Co doping	Co‐doped WS_2_	–	–	–	354.3 Oe	−5.393 to 15.681 eV	–	[44]
		Co‐doped WSe_2_	–	565.7 emu cm^−3^	181.2 emu cm^−3^	1.2 kOe (10 K)	–	–	[46]
	Ni doping	Ni‐doped WS_2_	–	–	–	79.91 Oe	–	–	[31]
		Ni‐doped MoS_2_	–	–	–	40 Oe	–	–	[36]
	Mn doping	Mn doped MoS_2_	80 and 150 K	–	–	1000 Oe	−4.93 eV	–	[30]
		Mn‐doped WS_2_	–	–	–	–	–	–	[44]
	V doping	V‐doped WS_2_	–	1 µ_B_	–	–	0.24 eV	Ferromagnetic state	[40]
		V‐doped MoS2	242 K	–	–	–	–	–	[39]
		V‐doped WS_2_	–	0.280 µ_B_	–	–	0.312 eV	–	[37]
	Rare earth doping	Dy‐doped MoS_2_	–	–	0.023 emu g^−1^	–	Semiconductor	Room‐temperature ferromagnetic	[52]
	Noble metal doping	Ni‐doped WS_2_	–	3.213 µ_B_	–	–	Half‐metallic	Ferromagnetism	[57]
		Pd‐doped WS_2_	–	2.841 µ_B_	–	–	Half‐metallic	Ferromagnetism	[57]
		Pt‐doped WS_2_	–	2.920 µ_B_	–	–	Half‐metallic	Ferromagnetism	[57]
		Ag‐doped WS_2_	–	0.817 µ_B_	–	–	Half‐metallic	Ferromagnetism	[58]
	Nonmetallic elements doping	H‐doped MoSe_2_	–	1.0 µ_B_	–	–	Half‐metallic	Ferromagnetism	[59]
		P‐doped MoSe_2_	–	1.0 µ_B_	–	–	Semiconductor	Ferromagnetism	[59]
		C‐doped MoSe_2_	–	0 µ_B_	–	–	Half‐metallic	Nonmagnetic	[59]
Structure regulation	Composites	MoS_2_/graphene	61 K	–	–	–	40 meV	–	[67]
		CrI_3_/WSe_2_ composites	300 K	–	–	–	0.9 eV	–	[69]
	Adsorption	Cr‐adsorbed	–	–	–	–	–	–	[67]
		MoS‐adsorbed	–	–	–	–	−0.23 eV	–	[70]
		Pt‐adsorbed MoSe_2_	–	–	0.66 µB	–	1.38 eV	–	[71]
	Phase regulation	VSe_2_	–	–	–	–	0.06 eV	Ferromagnetism	[75]
		MoSe_2_	–	0.027 µ_B_	–	–	54.9 eV	Ferromagnetism	[74]
	Size or edge regulation	AWS_2_NR‐3H	–	2 µ_B_	–	–	0.5 eV	Ferromagnetism	[76]
		zz‐MoS_2_‐NR‐Mo5	–	0.85 µ_B_	–	–	Metallic	Ferromagnetism	[81]
External field treatment	Lattice strain	MoTe_2_‐V_MoTe3_	–	12.02 µ_B_	–	–	1.10 eV	–	[23]
		WS_2_	–	2.01 µ_B_	–	–	0.13 eV	–	[90]
	Electron irradiation	MoS_2_	–	1.46 emu g^−1^ (0.042 µ_B_/Mo) (5 K)	–	0.1 kOe (5 K)	Semiconductor	Ferromagnetism	[08]
	O/N plasma	O_40_‐MoS_2_	–	–	0.0343 emu g^−1^ (300 K)	–	Semiconductor	Diamagnetic	[09]
	Electron injection	MX_2_ (M = Mo, W; X = S, Se, Te)	–	–	–	–	Semiconductor	Ferromagnetism	[89]

**Table 2:** Advantages and disadvantages of the ferromagnetic enhancement strategies for TMDs

Strategies	Methods	Materials	Advantages	Disadvantages	Refs.
Defect introduction	Vacancy	M vacancy	Compared with the perfect MX_2_, the M vacancies introduce impurity states, leading to asymmetric spin‐up and spin‐down DOS near the Fermi energy, and the induced local spin polarization leads to the formation of magnetic moments in the system. A larger magnetic moment can be induced by M vacancies compared to X vacancies.	Under the same conditions, compared with X vacancies, M vacancies have a higher formation energy and do not occupy a thermodynamic advantage. At present, the research on M vacancies mainly adopts computational simulation methods.	[22, 23]
		X vacancy	Under the same conditions, the introduction of X vacancies has a lower formation energy, and the introduction process is simpler. Moreover, the introduction form of X vacancies is more flexible (double X vacancies, interaction with doping elements, etc.), which can produce different magnetic regulation effects.	Due to the possible formation of M‐M metal bonds around the X vacancies, resulting in the disappearance of the unsaturated dangling bonds of X, the X vacancies are generally nonmagnetic in many TMDs systems. It needs to cooperate with various other regulation means to work together to generate magnetism.	[24, 25, 26]
	Doping	Fe doping	The iron‐doped molybdenum disulfide monolayer maintains magnetization, exhibits ferromagnetism at room temperature, and has potential long‐range room temperature ferromagnetic interaction and large magnetic moment.	The dependence of magnetic exchange coupling on the layer when iron is doped. Unlike the molybdenum disulfide monolayer, the tungsten disulfide monolayer does not exhibit a ferromagnetic magnetic phase transition when iron is doped. It does not have certain universality.	[27, 28, 29]
		Mn doping	The single‐doped selenium disulfide structure exhibits magnetic half‐metallic properties, that is, some energy levels in the spin‐up channel pass through Er, showing metal characteristics, while the spin‐down channel is still a semiconductor.	Manganese has an adverse effect on the surface functionalization of 2D materials in both alkaline and acidic media.	[30, 33, 34]
		Ni doping	The doped nickel atoms and other transition metal elements tend to stay in the nearest adjacent position at high concentrations, and ferromagnetic coupling occurs between them.	Nickel can only produce room temperature antiferromagnetic coupling with low spin structure.	[31, 35, 36]
		Cr doping	Chromium dopant can effectively improve the ferromagnetic behavior of molybdenum disulfide nanosheets.	When the doping concentration of chromium is high, the antiferromagnetic coupling is dominant, which weakens the ferromagnetism of the system. And the effect of chromium‐doped system on high frequency permeability is limited.	[37, 38]
		V doping	Vanadium doping can produce good diluted magnetic semiconductors. And it can induce ferromagnetism at very low vanadium concentration.	The vanadium material introduced at room temperature is metastable, and annealing above 500 K will form a thermodynamically favorable impurity configuration, showing reduced ferromagnetism.	[39, 40, 41]
		V, Nb, Ta codoping	The doping system exhibits p‐type doping and metal characteristics, and the system also induces ferromagnetism.	Although the doping system maintains the original geometry of the original tungsten disulfide monolayer, there is also a slight lattice distortion.	[42, 43]
		Co doping	The induced spin on the nearby main atom is antiparallel to the magnetic spin of the doped atom, and the doping of cobalt can obtain a higher total magnetic moment.	Most of the samples have a wide variety of sulfides, oxides and metal phases during the reduction process, resulting in morphological defects.	[44, 45, 46]
		Cu doping	Although the Cu itself does not have magnetism, the Cu‐doped TMDs have a large spin magnetic moment, that is, Cu can introduce strong iron magnetism. At the same time, the Cu doping can get a higher Curie temperature.	Cu doping is sensitive to concentration changes, and excessive Cu doping may cause interactions between Cu atoms, resulting in a decrease in the magnetic moment. At the same time, Cu atoms are easy to migrate or diffuse, resulting in poor system stability.	[47, 48, 49, 50]
		Rare earth doping	Rare earth dopants with unfilled 4f and 5d energy states and abundant electronic energy levels can provide strong spin–orbit coupling to tune the semiconducting properties of TMDs materials.	Rare earth dopants are costly and polluting. At the same time, the higher activity of rare earth elements also makes it possible to generate impurity phases during the doping process.	[52, 53, 54, 55, 56]
		Noble metal doping	The formation energy of noble metal doping is low. Noble metal doping can stably enhance the magnetic moment, and the magnetism of noble metal‐doped TMDs can be controlled by strain, which provides a new idea for spintronic devices.	Noble metals are expensive, and currently the types of TMDs suitable for noble metal doping are relatively single.	[57, 58]
Structure regulation	Composites	CrI_3_/WSe_2_ composites	It broadens the capabilities of magnetic interface design for spintronic applications and provides powerful and efficient spin transport.	The absence of bandgaps and ferromagnetic ordering in many materials severely limits their use in spintronics.	[69]
	Adsorption	NbSe_2_ adsorption	Surface molecular adsorption will be a powerful tool for regulating spin ordering in 2D materials.	The performance of electronic devices modified by this means is highly dependent on the morphology of the defects and the quality of the low‐dimensional material, which tends to cause magnetic ion aggregation.	[72]
	Phase regulation	VTe_2_ phase regulation	This modification means that the ferromagnetic (FM) ground state is stable under ambient conditions, offering amorphous nature as a new option for tailoring TMD magnetization for future spintronic applications.	The appropriate temperature between the different structural phase transitions affects the electronic structure and magnetic properties of the intermediate stages of the structural phase transition, with harsh modification conditions.	[78]
	Size or edge regulation	The passivating the ribbon edges with H, C and so on of WS_2_NRs, MoSe_2_, MoS_2_	After edge passivating, they exhibit different structural stabilities and electronic structures according the patterns of edge passivating.	The edge activity of bare edge is very high due to dangling bonds of edge atoms. Other atoms were absorbed to edge atoms with different ways. Therefore, the experimental operation process is complicated.	[76, 79, 80, 81, 82]
		Different annealing methods of magnetron sputtering improve the crystallinity of materials, thereby inducing changes in ferromagnetism	Sputtering is a promising method for inducing strong‐edge ferromagnetism, and improving the crystallinity, while controlling *µ*_s_ is very important.	The cost of laboratory equipment is high.	[77]
External field treatment	Lattice strain	MoTe_2_, MoS_2_, WS_2_, WSe_2_	Tuning the bandgap and inducing spin polarization without introducing impurity atoms.	Strain engineering induces magnetic properties, mainly for structural defects or doping.	[7, 23, 84, 85, 86, 87]
	Electron irradiation	MX_2_ (M = Mo, W: X = S, Se, Te)	It is simpler than chemical method and can effectively induce large magnetic moment.	High cost and complex experimental conditions.	[8, 9, 88, 89]
	O/N plasma				
	Electron injection				

## 7 Summary and Outlook

In summary, magnetic modulation of transition metal disulfide with excellent properties in the field of optoelectronics has been reviewed. The existence of conventional magnetic ions may not be necessary for the magnetism of the transition metal disulfide, but the local magnetic moment can effectively regulate the magnetism of the transition metal disulfide. It mainly involves transition metal doping, vacancy defects, heterostructure recombination, phase modulation, dimensional edge modulation, and adsorption. Metal doping is widely used. Some nonmagnetic Cu, Mn, rare earth elements, and precious metals can effectively induce the magnetic properties of TMDs by doping. The vacancy defect mechanism often introduces vacancies such as Mo, S, and Se, so that unpaired electrons appear around the material, which provides magnetic distance to the system and significantly changes the charge distribution, thus inducing ferromagnetism. Compared with TMDs monolayers doped with metal alone, the composite heterostructures exhibit stronger magnetic moments and a more stable ferromagnetic state, with stronger adsorption capacity and better photocatalytic efficiency. Phase modulation affects the electronic structure and magnetic energy at the intermediate phase of phase transition through the appropriate temperature between different structural phase transitions. Size or edge modulation is the addition of unsaturated atoms at edge positions by induction of defects (vacancies, dislocations, grain boundaries) or absorption of nonmetals (such as H, O, C, B, N, and F), resulting in magnetism. In addition to the above common methods, it can also be achieved by electron irradiation induction, O‐plasma processing, applied electric field, and electron (hole‐free) injection.

However, the magnetic properties of 2D TMDs still have many limitations and challenges for industrial applications. The first is the efficient doping technique of elements at specific positions on the TMD. Due to the very thin structure of TMDs, the atomic composition is more complex than graphene, which increases the difficulty of doping ions at specific locations. Therefore, commonly used injection methods are not suitable for TMDs doping. More efficient and reliable doping techniques, such as the insertion of layers of impurity atoms between 2D materials, are deserved to be subsequently developed. The second is the industrial‐scale preparation technology of core devices. For 2D heterostructures that have natural advantages in stacked devices without layer–layer lattice‐misfit issues, the flexible assembly of layer‐by‐layer heterostructures can also enable electronic/optoelectronic devices with novel properties and more competitive performance. However, their preparation is more difficult than pure TMDs due to the complexity of structures and the based devices. Improved methods such as CVD should be vigorously developed for the scalable preparation of functional heterogeneous structures. The third is the performance stability of the device. Since pure 2D magnetic materials are usually unstable in air, 2D TMDs doped with magnetic atoms have relatively stable structures and can form 2D dilute magnetic semiconductors. Modulating the ferromagnetism of TMDs materials is still in the fundamental stage of spintronic device development, and subsequent studies are expected to explore the design of novel heterojunctions by combining thin magnetic semiconductors, antiferromagnetic semiconductors, and superconductors to develop next‐generation ferromagnetic devices. In conclusion, research and development related to ferromagnetic 2D materials still require significant investment to help achieve their large‐scale commercial applications.

## Conflict of Interest

The authors declare no conflict of interest.
